# Combination of Chloroform with Opiates for the Relief of Pain

**Published:** 1869-12

**Authors:** 


COMBINATION OF CHLOROFORM WITH OPIATES FOR THE RELIEF OF PAIN.—Dr. W. Marshall strongly recommends (*Glasgow Medical Journal*, May, 1869) this union of remedies for anodyne purposes. From 10 to 20 minims of chloroform are combined with one or two drachms of compound tincture of camphor (if the pain be moderate), or 10, 20, or 40 minims of Battley's sedative (if it be severe). This generally produces sleep within a few minutes, and its effects are more lasting than those of an opiate alone, and without its disagreeable after-effects. It ought to be given in some thickish solution, such as mucilage; otherwise the chloroform will fall to the bottom. —*The Practitioner.—Richmond and Louisville Med. Journal.*

